# Effectiveness of in-Line Filters to Completely Remove Particulate Contamination During a Pediatric Multidrug Infusion Protocol

**DOI:** 10.1038/s41598-018-25602-6

**Published:** 2018-05-16

**Authors:** Maxime Perez, Bertrand Décaudin, Wadih Abou Chahla, Brigitte Nelken, Laurent Storme, Morgane Masse, Christine Barthélémy, Gilles Lebuffe, Pascal Odou

**Affiliations:** 1 University Lille, EA 7365 – GRITA – Groupe de Recherche sur les Formes Injectables et les Technologies Associées, F-59000 Lille, France; 2 Institute of Pharmacy, CHU Lille, F-59000 Lille, France; 3 Department of Pediatric Hematology, Jeanne de Flandre Hospital, CHU Lille, F-59000 Lille, France; 4 Department of Neonatology, Jeanne de Flandre Hospital, CHU Lille, F-59000 Lille, France; 5 University Lille, EA 4489 – Environnement Périnatal et Santé, F-59000 Lille, France; 6 Department of Anesthesia and Intensive Care Medicine, CHU Lille, F-59000 Lille, France

## Abstract

The large number of drugs administered simultaneously to neonates and children in hospital results in the formation of particles that are potentially infused. We have investigated the ability of IV in-line filters to eliminate particulate matter from multidrug infusion lines and so prevent contamination. The impact on particle occurrence of the internal volume of the IV line below the in-line filter was then evaluated. The multidrug therapy given to children was reproduced with and without in-line filtration. Three combinations with a filter were tested to vary the internal volume (V) between the filter and the catheter egress. The catheter was then connected to a dynamic particle count to evaluate the particulate matter potentially administered to children during infusion. The introduction of in-line filters led to a significant reduction in overall particulate matter, from 416,974 [208,479–880,229] to 7,551 [1,985–11,287] particles (p < 0.001). Larger particles of ≥10 and 25 µm were also significantly reduced. Adding an extension set to the egress of the in-line filter (V = 1.7 mL) caused a significant increase in particulate contamination for both. This study showed that in-line filtration is an effective tool in preventing particle administration to patients. Their position in the infusion in-line is therefore important because of its impact on internal volume and drug particle formation.

## Introduction

Intravenous (IV) therapy is commonly used in neonatal and pediatric patient populations. They receive many drugs simultaneously through a limited number of venous accesses, which contribute to the risk of drug-drug interactions leading to precipitates. Moreover, contamination of IV fluids can be associated with several contaminants, such as microorganisms, particles or air bubbles. In intensive care units (ICUs), patients can receive up to one million particles daily^1^. We recently conducted a study to assess the effect of infusion sets on overall particulate contamination exposure during multidrug IV therapy in a pediatric hematology ward^2^. We showed that, under specific conditions, the use of a multi-lumen infusion set helped to reduce this contamination. The study also indicated however that patients can receive up to 900,000 particles per day during hospitalisation despite these infusion systems, which caused us to turn our attention to in-line filtration. Neonatal and pediatric populations receiving a great number of particles, especially large ones, are particularly affected as shown in a previous study^3^. These may cause serious damage to the body, especially large particles of about 10 µm in diameter^4^. Images of particles and conglomerates of considerable size (40 × 20 µm) on the surface of various in-line filters were displayed^5^. Clinical complications may include injection site reactions, i.e. phlebitis^6^, granuloma^7^, arterial embolism, modulation of immune response, microcirculation deterioration^8^, and even death^7,9^.

Preventing the infusion of particles to the body is therefore a priority, especially for critically ill patients (i.e. neonate and pediatric populations). A few studies have demonstrated that, under specific conditions, infusion device characteristics could have an impact on preventing physical drug incompatibilities^10,11^. Nonetheless, even after infusion through multi-lumen catheters, intravascular precipitation has been reported^12^, indicating that the risk of drug precipitation cannot be completely avoided.

IV in-line filters seem to offer the advantage of preventing particulates, micro-organisms and air from being infused. Despite insufficient evidence to recommend their use to prevent morbidity or mortality in neonates^13^, infusion-related phlebitis^14^ or the incidence of sepsis^15^, several studies have shown they can be useful for critically ill patients. A single-centre prospective and randomised controlled trial (RCT), including 88 newborn infants, found a significant reduction in major complications, such as thrombi and clinical sepsis, compared to the control group^16^. More recently, a pediatric RCT showed that in-line filters resulted in a significant decrease in overall complication rates and the incidence of systemic inflammatory response syndrome (SIRS)^17^. The authors also showed that the duration of mechanical ventilation and hospitalisation were reduced in the filter group. In the same RCT, in-line filters could significantly reduce respiratory, renal and hematological complications^18^. Filters had no effect on the occurrence rates of cardiovascular, hepatic and neurological dysfunctions from one group to the other. The advantage of in-line filtration was analysed in a subgroup of cardiac patients, showing significant decreases in the occurrence of SIRS, renal and hematological complications compared to a control group.

In some studies no benefit was to be found from using in-line filters in infusion sets. In a recent RCT including critically ill adult patients, the authors did not find any significant differences in the number of ICU days with SIRS^19^. They even showed a higher incidence of SIRS in the in-line filter group.

Brotschi *et al*. demonstrated, in an *in vitro* study, that in-line filters reduce flow irregularities during IV infusion^20^. The use of filters has become the subject of recommendations, especially for parenteral nutrition (PN). Two types of filters are recommended: 0.2-µm filters for clear PN (i.e. binary PN), and 1.2-µm filters for lipid-containing PN^21–25^.

Few studies have assessed particulate matter in IV drugs or fluids after filtration by calculating the number of particles contained in different lyophilised preparations (for example ceftriaxone) with and without filtration^26^. As expected, the use of filters resulted in a reduction of about 96% in particulate matter. Other studies have indirectly evaluated the particulate matter in IV therapies by analysing filtration membranes^5^.

The purpose of this study was to assess the ability of IV in-line filters to eliminate particulate contamination in multidrug infusions, including highly incompatible drugs, and consequently evaluate the impact of the internal volume of the IV line below the in-line filter on the occurrence of particles.

## Results

**Figure 1:**
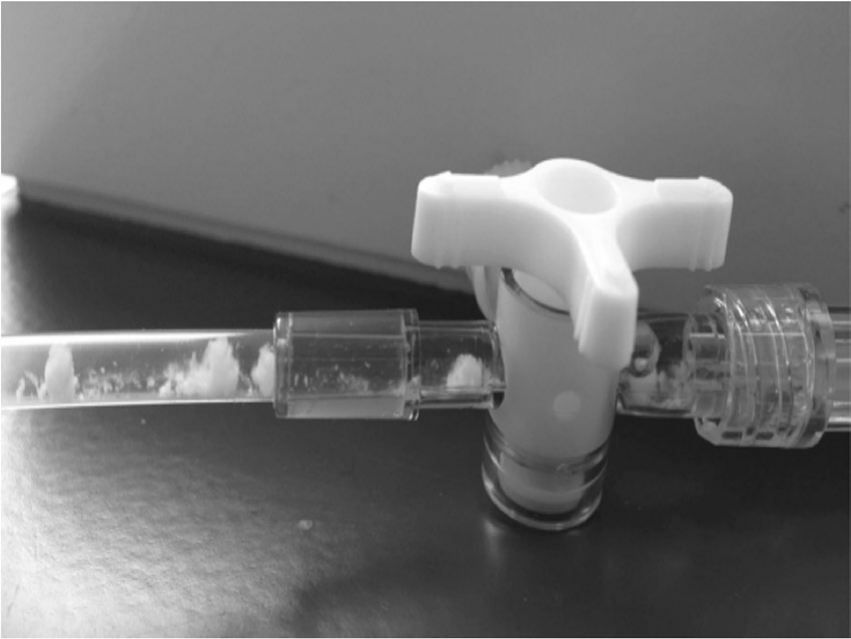
Visible precipitate observed at the egress of the 3-way stopcock of the IV infusion line due to physical drug-drug incompatibility. It was noted that drug flocculates are not visible to the human eye at the catheter site.

As expected, a visible and intense precipitate was observed in the IV infusion line during the infusion of the pediatric multidrug protocol, due to the presence of drug-drug incompatibilities (Fig. 1 and Video 1). This precipitate occurred upstream of the infusion line and gradually disappeared along the infusion line. Despite the absence of visible precipitate at the distal end of the IV infusion line, sub-visible particles could be detected by the dynamic camera (Video 2).

**Figure 2:**
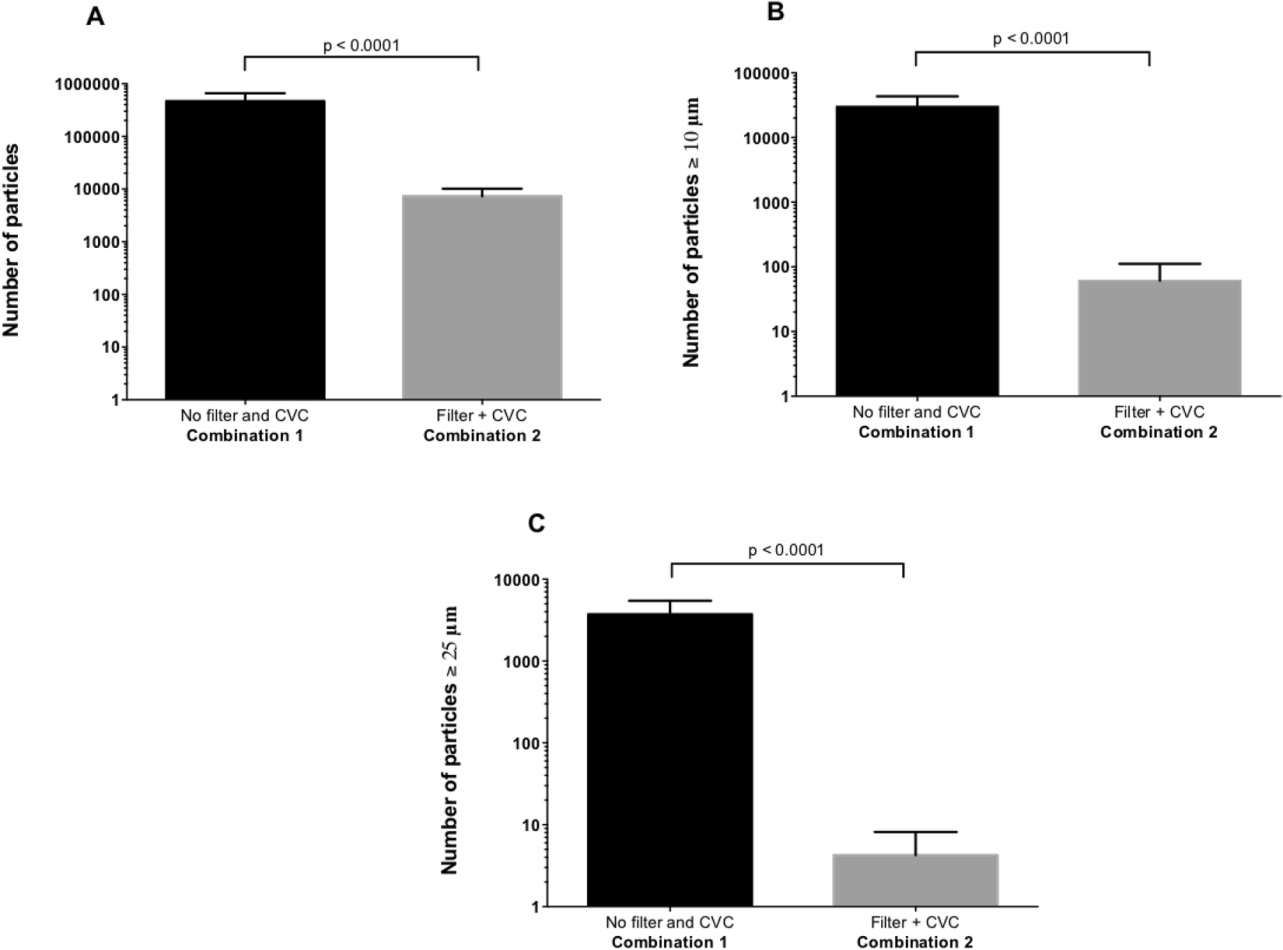
Number of particles measured at the egress of the catheter without and with the in-line filter: overall particulate matter (Fig. 3A), particles ≥10 µm (Fig. 3B) and 25 µm (Fig. 3C). The Y-axis is plotted on a logarithmic scale for all histograms.

An IV in-line filter directly connected to the central venous catheter (CVC) significantly decreased overall particulate contamination during the 24-hour infusion period compared to infusion without filter (Table 1 and Fig. 2). Figure 2B,C also show that filtration significantly reduced the number of particles, sizes ≥10 µm and 25 µm, respectively.

**Table 1:** Comparison of particulate matter between infusion combinations with and without IV in-line filtration.

Infusion combinations	Combination 1 “Without filter and with CVC”	Combination 2 “With filter and CVC”	Adjusted *p*-value
Total number of particles at T24	416,974 (208,479–880,229)	7,551 (1,985–11,287)	<0.0001
Particulate matter ≥10 µm	29,340 (9,921–51,097)	43 (7–150)	<0.0001
Particulate matter ≥25 µm	3,458 (1,201–6,927)	3 (0–11)	<0.0001

The total number of particles was assessed after 24-hour multidrug administration (N = 10).

**Figure 3:**
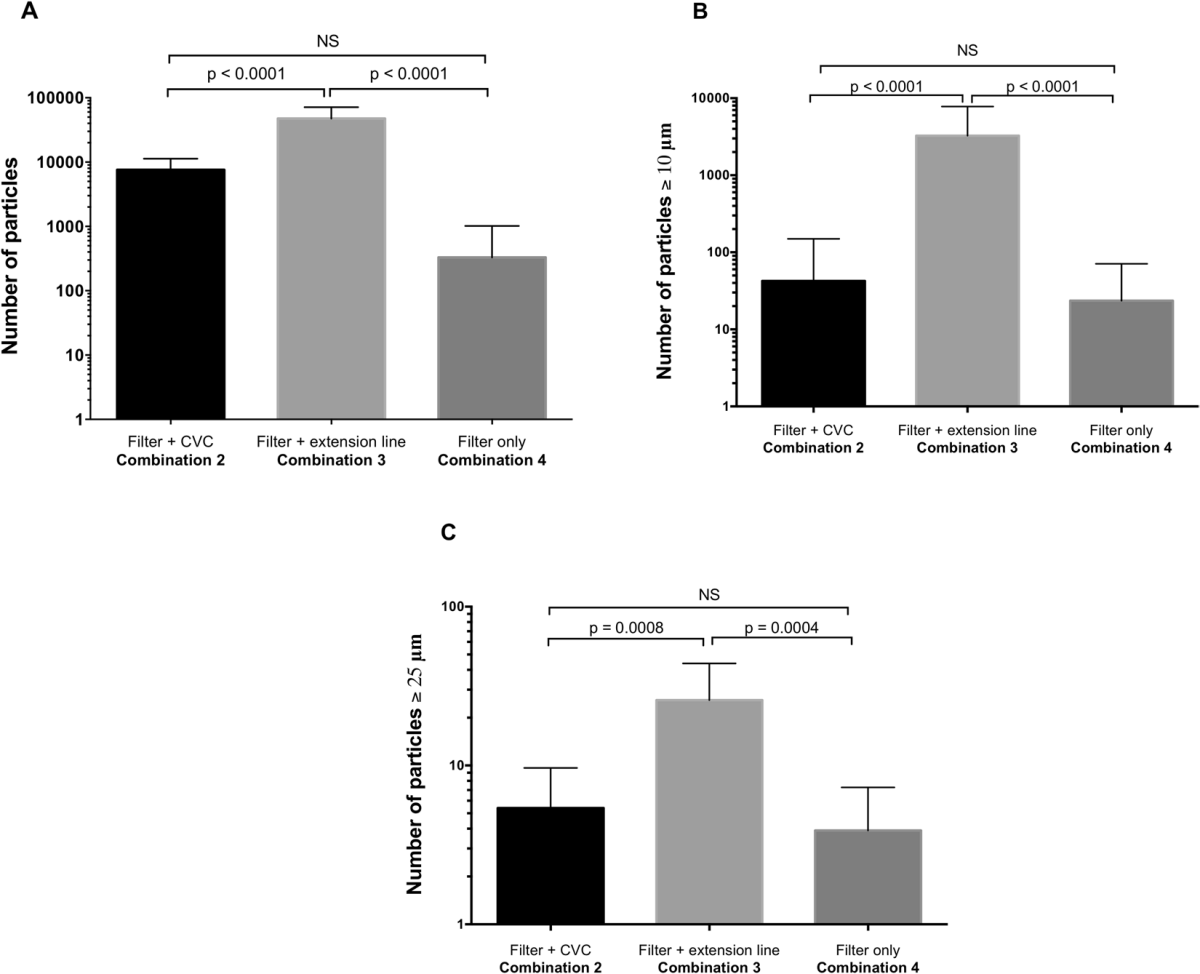
Comparison of quantified particulate matter between the 3 combinations with in-line filtration, focusing on overall particulate matter (Fig. 4A), particles ≥10 µm (Fig. 4B) and 25 µm (Fig. 4C). The Y-axis is plotted on a logarithmic scale for all histograms.

For both total particles and the larger ones, particulate contamination was not significantly different between the egress of the filter (combination 4 “with filter only”; V = 0 mL) and the egress of the catheter directly connected to the filter (combination 2 “with filter and CVC”; V = 0.8 mL) (Table 2 and Fig. 3).

**Table 2:** Comparison of particulate matter in all infusion combinations including the addition of IV in-line filtration.

Infusion set combination^*^	Particulate contamination analysis	Mean difference	95% confidence interval of difference	Adjusted *p*-value
Combination 2 “with filter and CVC” *vs*. combination 4 “with filter only”	Overall particulate matter	6,887	−6,761 to 20,536	0.4343
	Particulate matter size ≥10 µm	29	−1,240 to 1,298	0.9982
	Particulate matter size ≥25 µm	2	−11 to 14	0.9495
Combination 3 “with filter + extension line” *vs*. combination 4 “with filter only”	Overall particulate matter	43,442	29,793 to 57,090	<0.0001
	Particulate matter size ≥10 µm	−3,513	−4,782 to −2,244	<0.0001
	Particulate matter size ≥25 µm	22	10 to 34	0.0004
Combination 2 “with filter and CVC” *vs*. combination 3 “with filter and extension line”	Overall particulate matter	−36,554	−50,203 to −22,906	<0.0001
	Particulate matter size ≥10 µm	3,513	2,244 to 4,782	<0.0001
	Particulate matter size ≥25 µm	−20	−33 to −8	0.0008

^*^Infusion set combinations are represented by the volume V, corresponding to the internal volume of the IV tubing between the in-line filter and the Qicpic particle counter, i.e. V = 0.8 mL, 1.7 mL and 0 mL for combinations 2, 3 and 4, respectively.

Adding an extension set to the egress of the in-line filter (combination 3 “with filter and extension line”; V = 1.7 mL) caused a significant increase in particulate contamination for both particles as a whole and the larger ones (Table 2 and Fig. 3).

**Figure 4:**
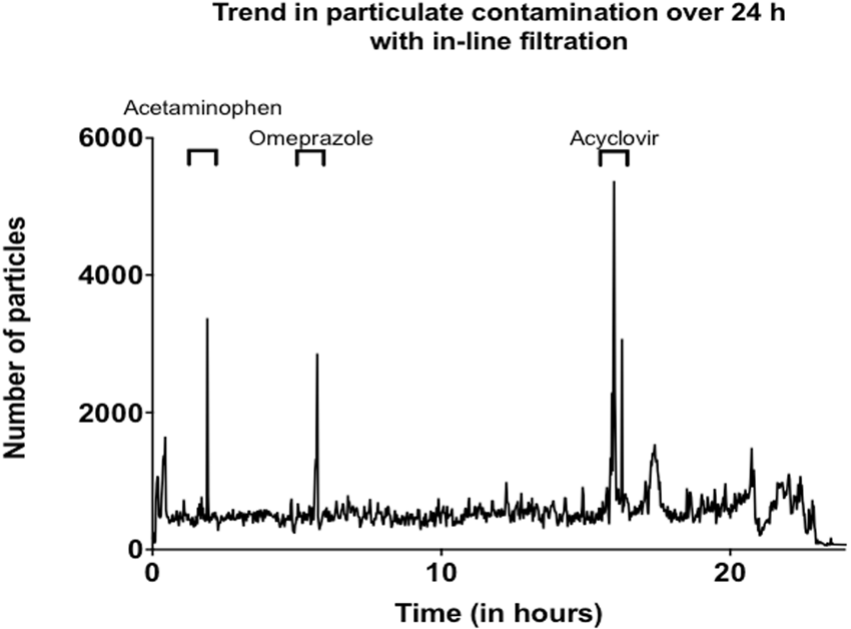
Trend in particulate contamination below the in-line filter over a 24-hour period. Peaks observed in the figures are based on discontinuous administrations of drugs during the pediatric multidrug protocol.

Figure 4 shows that the administration of discontinuous drugs (i.e. acetaminophen, omeprazole and acyclovir) leads to disturbances in particulate contamination. Each intermittent IV infusion resulted in the infusion of particle bolus.

## Discussion

Theoretically, no particles should be detected in the infusion line following in-line filtration. Despite filtration however, drugs are still in contact inside the CVC, which could account for the particles detected. The standard infusion line including a filter and a CVC directly connected to it was seen to be associated with more than 7,000 particles infused per day. Decreasing the internal volume of the IV line below the in-line filter by removing the CVC resulted in a substantial decrease in the total number of particles whereas the addition of a 3-way stopcock with 25-cm infusion line (i.e. 1.70 mL) significantly increased overall particulate matter. These results suggest that the dead-space upstream of the IV in-line filter (i.e. corresponding to CVC volume), contributed to facilitating drug contact inside the CVC, resulting in the presence of sub-visible particles especially when infusing incompatible drugs. Likewise, our results showed differences for larger particles, ≥10 µm and 25 µm, from one combination to another, with or without CVC, with a significant decrease when a filter was added. The increase in internal volume when using a 3-way stopcock with its infusion line also significantly increased the number of larger particles. It has been hypothesised that the formation of larger particles requires prolonged contact time between drugs in the internal volume of the IV line below the in-line filter. Our study highlights the fact that in-line filters should be added, as far as possible, at the egress of each infusion set.

Our results are in accordance with a previous study that showed that, despite the gradual blackening of in-line filters during PN bag infusion, the filter membrane continued to filter solutions efficiently for up to 72 hours with an acceptable rate of released particles^27^. Few studies have assessed particulate matter in IV drugs or fluids after filtration. Kuramoto *et al*., mentioned above, evaluated the number of particles contained in different lyophilised preparations with and without filtration and showed, as expected, that filters produced a reduction of about 96% in particulate matter^26^. Other studies have indirectly evaluated the particulate matter in IV therapies by analysing filtration membranes^5^. This study however is the first to demonstrate that zero threshold of particulate contamination does not exist in real and dynamic infusion conditions. The use of in-line filters helps to significantly reduce overall particulate contamination during drug infusion but whatever the infusion conditions, we have to consider that patients will receive particles even with the use of in-line filters. The objective now is to reduce this particulate contamination as far as possible, especially by limiting delivery interaction between co-infused medications (i.e. by adapting infusion flow rate, concentration, separation of incompatible drugs, etc.) so as to minimise drug contact inside the CVC. In this context, the use of specific multi-lumen infusion devices associated with filters may be the solution^2,10,11^.

On the basis of our study findings, particles <10 µm represent more than 99% of the particles counted. Although they have a smaller diameter than pulmonary capillaries (i.e. 2 µm to 15 µm), these sub-visible particles are not devoid of toxicity for the body. About one third of the injected microparticle dose may be localised in the lungs. Similarly, Ilium *et al*. showed that small particles can also diffuse into various organs such as the liver or spleen, leading to deleterious effects on their functions^4^. Larger particles >10 µm were also counted during IV filtered infusions. In studies cited above, it has been shown that these particles were trapped in the lungs, whatever their nature, leading to obstruction of the pulmonary vessels. Recently, a German research group assessed the clinical effects of particulate contamination during IV drug infusion into the human body^17,18,28^. Delivery of particles was responsible for an increase in the incidence of SIRS, deleterious effects on microcirculation and organ failures.

However, there are limitations to this study. The particulate counter analysis has a detection limit of 1 µm, which probably underestimates the number of particles really administered to patients. It is quite conceivable that a large proportion of particles of between 0.2 µm and 1 µm have been disregarded in overall particulate matter. Furthermore, experiments were performed in static conditions, without any disturbance along the infusion line. As demonstrated in our previous study^2^, this condition favours a basal level of particulate contamination, whereas it is known that any disturbance to balance (especially changes in drug or fluid flow rates) may affect particle exposure. This study shows that, despite in-line filtration, particles may be administered to patients. No chemical analysis was performed to identify the nature of the precipitate which is probably the result of incompatibility between vancomycin and piperacillin-tazobactam, as already observed in our experience and mentioned in a previous publication^2^. Nevertheless, it is necessary to take into account the presence of other drugs which are potential vectors of incompatibility (i.e. acyclovir or omeprazole). It would be interesting to specify the nature of particle exposure so as to analyse the effects of drug-drug interaction or identify specific particles of glass or plastic. Chemical analysis could also determine any interactions between drugs and filter membrane, in relation to drug retention. A limited number of cases concord with this^29^, whereas most studies have revealed no variations in drug concentration when using in-line filters^30–33^. Room temperature should also be taken into account because of its impact on particle formation.

In conclusion, this study is the first step towards evaluating particulate matter in pediatric multidrug IV therapy with an in-line filter, especially when infusing two incompatible drugs that lead to precipitate. It validates the concept that in-line filtration is an effective tool in limiting particle administration to patients, but it is not sufficient to eliminate particle infusion. Particulate contamination is never non-existent notably when using an infusion line where the internal volume of the IV line below the in-line filter is high. This study demonstrates that the in-line filter should be positioned as close as possible to the patient to be most effective by minimising internal volume. It would therefore be appropriate to set in motion the means required to establish a clinical protocol.

## Methods

### Pediatric Multidrug Infusion Protocol

This study exactly reproduced the conditions of drug use during therapy administered to children receiving hematopoietic stem cell transplantation for leukemia, in our pediatric ward at the Lille University Hospital (France).

All components were standard clinical materials and devices. Solutions and medications were prepared according to manufacturers’ instructions (Table 3). In our laboratory the infusion set was replicated and consisted of a standard single-lumen IV infusion set, with a 4-port manifold and 150-cm extension line (ref. RPB4320, 14A20-T, Cair LGL, Lissieu, France). The carrier fluid consisted of a solution of 5% glucose with electrolytes (3 g/L hypertonic saline solution, 2 g/L potassium chloride and 10% 3 g/L magnesium chloride). All medications and fluids were infused via an in-line 0.22 µm pore size filter (ref. AEF1NTE, 14–658; Pall Medical, Fribourg, Switzerland). Piperacillin-tazobactam, acetaminophen, acyclovir and the carrier fluid were infused with a volumetric pump (MVP module Orchestra, Fresenius Vial, Brezins, France). Vancomycin, cyclosporine-A and omeprazole were infused through syringe pumps (DPS module Orchestra, Fresenius Vial, Brezins, France). All extension lines were flushed with the carrier fluid before use, as is routinely performed by nurses.

**Table 3:** Description of tested drugs.

Drug	Dosage (per 24 h)	Solvent for reconstitution/dilution	Final concentration (mg/mL)	Infusion flow rate (mL/h)	Time of infusion (duration)
Vancomycin HCl (Sandoz)	2 g	Water for injection	41.67	2	Over 24 h
Piperacillin Na – Tazobactam (Mylan)	14 g	Water for injection/5% glucose	116.67	5	Over 24 h
Cyclosporin-A (Sandimmun, Novartis)	60 mg	Saline solution	1.25	2	Over 24 h
Acetaminophen (BBraun)	600 mg	Water for injection	10.00	120	T0 + 2 h (30 min)
Omeprazole (Mylan)	40 mg	Saline solution	2.00	20	T0 + 5.5 h (30 min)
Acyclovir^*^ (Zovirax, GlaxoSmithKline)	400 mg	Water for injection/saline solution	8.00	80	T0 + 16 h (1 h)
*Carrier fluid*		—		40	Over 24 h
Glucose	5 g/dL		0.5		
Saline solution	3 g/L		0.03		
Potassium chloride	2 g/L		0.02		
Magnesium chloride	3 g/L		0.03		

^*^Saline rinsing was performed before (11.25 min) and after (11.25 min) the infusion of acyclovir (2 × 15 mL) at the same infusion flow rate as the drug (80 mL/h).

**Figure 5:**
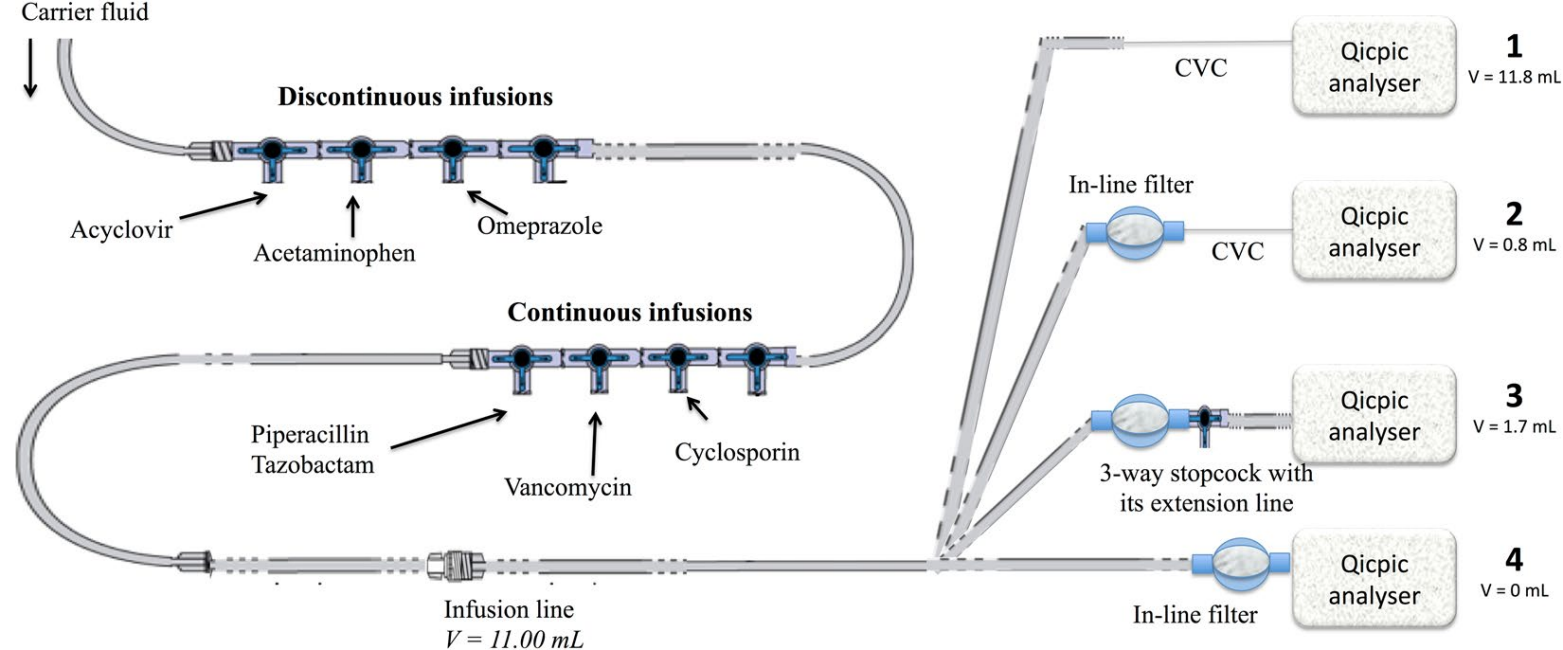
Schematic representation of the infusion line used in the pediatric department. The carrier fluid (i.e. 5% dextrose with electrolytes) was infused through a first 4-stopcock set dedicated to discontinuous drug infusions (acyclovir, acetaminophen and omeprazole). The infusion set was connected to a 150-cm extension line, connected to a second 4-stopcock set for continuous drug infusions (i.e. piperacillin/tazobactam, vancomycin and cyclosporin). A 350-cm extension line was then added to the infusion line (V = 11.00 mL). The infusion line was finally connected to the Qicpic instrument, with or without the central venous catheter (CVC). For all experiments, the infusion flow rate of the carrier fluid was 40 mL.h^−1^.

Four measuring locations and combinations were compared (Fig. 5):

1. At the egress of the catheter of a standard infusion set and catheter as described in a previous study (2), with a CVC and without any IV filter (i.e. combination 1 “without filter and with CVC”).
2. At the egress of the catheter of an infusion line with a Broviac 6.6-fr single-lumen CVC, length = 57 cm; internal diameter = 1.0 mm (ref. 0600540, HUXE0422, Bard, Salt Lake City, Utah), added directly after the in-line filter (internal volume of the IV line below the in-line filter V = 0.80 mL; i.e. combination 2 “with filter and CVC”).
3. At the egress of an infusion line with a 3-way stopcock and a 25-cm extension set connected directly after the in-line filter (V = 1.70 mL; i.e. combination 3 “with filter and extension set”).
4. At the egress of the in-line filter. In this case, the IV in-line filter was directly connected to the particle counter by removing the CVC (V = 0 mL; i.e. combination 4 “with filter only”).

### Analysis of particulate matter

All drug combinations were tested with extemporaneous mixtures. A visual inspection of the extension line against a black background and an obscured-light sub-visible particle count test were made to highlight the model of drug incompatibility.

For all experiments with and without in-line filter, the distal end of each IV administration set was directly connected to the Qicpic analyser through Luer-lock connections (Sympatec Inc., Clausthal-Zellerfeld, Germany). The internal volume of the Qicpic instrument was about 1 mL. The particulate counter dynamically detected all particles infused during therapy over a 24-hour period.

Experimental infusion was repeated ten times for each combination, over 24 hours. Visual inspection of the IV infusion line was performed to detect drug-drug incompatibility. For all combinations, particle size analyses were based on overall particulate contamination plus larger particles (i.e. 10 µm and 25 µm), as defined by the European Pharmacopoeia^34^. All experiments were made at 18 ± 2 °C ambient temperature.

Comparing the number of particles infused over a 24-hour period with measuring combinations 1 and 2 indicates the ability of in-line IV filters to eliminate particulate contamination during multidrug infusion. Comparing the data obtained with measuring combinations 2, 3 and 4 assesses the impact on particle occurrence of the internal volume of the IV line below the in-line filter.

The normality of all data collection was assessed by the D’Agostino & Pearson test (*p* value >0.05). The Student t test was used to compare the number of particles obtained with combinations 1 and 2. Values obtained with combinations 2, 3 and 4 were compared using an analysis of variance (ANOVA). When this revealed a significant *p* value (*p* < 0.05), an analysis using Tukey’s test was performed to detect significant differences between couples of infusion sets. All statistical tests were performed using GraphPad Prism version 6.00 for MacOS X (GraphPad Software, La Jolla California USA, www.graphpad.com). Data is presented as median values and range (min – max), if not otherwise specified. All histograms are presented as mean values and standard error of the mean. For all analyses, statistical significance was considered as a *p*-value <0.05.

## Electronic supplementary material

Drug-drug incompatibility leading to the formation of a precipitate upstream of the infusion line

Detection of particles at the egress of the catheter by the Qicpic instrument
